# Three-Dimensional Block Matching Using Orthonormal Tree-Structured Haar Transform for Multichannel Images

**DOI:** 10.3390/jimaging6020004

**Published:** 2020-02-11

**Authors:** Izumi Ito, Aleksandra Pižurica

**Affiliations:** 1 Information and Communications Engineering, Tokyo Institute of Technology, Tokyo 152-8552, Japan; 2 Department Telecommunications and Information Processing, Ghent University, 9000 Gent, Belgium; Aleksandra.Pizurica@UGent.be

**Keywords:** block matching, Haar transform, multichannel image, multispectral image, color image

## Abstract

Multichannel images, i.e., images of the same object or scene taken in different spectral bands or with different imaging modalities/settings, are common in many applications. For example, multispectral images contain several wavelength bands and hence, have richer information than color images. Multichannel magnetic resonance imaging and multichannel computed tomography images are common in medical imaging diagnostics, and multimodal images are also routinely used in art investigation. All the methods for grayscale images can be applied to multichannel images by processing each channel/band separately. However, it requires vast computational time, especially for the task of searching for overlapping patches similar to a given query patch. To address this problem, we propose a three-dimensional orthonormal tree-structured Haar transform (3D-OTSHT) targeting fast full search equivalent for three-dimensional block matching in multichannel images. The use of a three-dimensional integral image significantly saves time to obtain the 3D-OTSHT coefficients. We demonstrate superior performance of the proposed block matching.

## 1. Introduction

Block matching is a fundamental tool used to search for blocks (patches) similar to a given query. It has been widely used in solving various image processing problems, like object recognition and tracking [1], image registration [2], image analysis [3], image restoration [4], to name a few. Block matching searches for patches of the same size as a query and is sensitive to deformation. In this sense, it is different from some common image descriptors such as scale-invariant feature transform (SIFT) [5] and speed-up robust features (SURF) [6], which extract features robust to deformation. In block matching, generally, a full search (FS) algorithm that exhaustively compares all the pixel intensities of all the candidates overlapping each other is the most accurate, but requires vast computation due to the huge number of candidates in a large search space. The larger the image size and number of image bands, the harder it is to use FS.

Fast block matching has been studied from algorithms and architectures. There are several works of architecture such as hardware acceleration and configuration by custom instructions specially for motion estimation in video coding [7,8,9,10,11]. There are also two categories in the algorithms, FS-equivalent algorithms and non-FS-equivalent algorithms. Some non-FS-equivalent algorithms such as three-step-search [12] and diamond search [13] reduce the amount of computation by limiting search areas, and others do so by approximating patterns [14,15], where there is a trade-off between accuracy and efficiency. The scope of this paper is limited to the FS-equivalent algorithm.

Fast FS-equivalent algorithms have been intensively developed in order to address the computational complexity with FS [16,17,18,19]. Although these methods can be applied only to the patches of size power-of-2, they prove that the search in a transformed domain is much more efficient than the search in a spatial domain. The additional calculation to obtain the transform coefficients is fully compensated by the reduction of candidates. The orthogonal Haar transform (OHT) reportedly performs fastest in this setting [20,21]. One of the reasons is that there is a unique way to calculate the transform coefficients using an integral image [22,23]. Once an integral image is built, the transform coefficients can be calculated with a few arithmetic operations. This is based on the fact that the Haar transform matrix is sparse and composed of rectangular functions, which is much different from other transforms. In order to overcome the limitation of patch sizes, two-dimensional orthonormal tree-structured Haar transform (2D-OTSHT), the generalization of OHT, was proposed [24]. Compared to OHT, the normalization factor of 2D-OTSHT is not an integer number, but this has little effect in the whole speedup.

In this paper, we propose three-dimensional orthonormal tree-structured Haar transform (3D-OTSHT) with three-dimensional integral image for multichannel images. In the proposed 3D-OTSHT, the transform coefficients can be obtained by a few arithmetic operations regardless of patch size. Focusing on the pruning performance in the transformed domain, we consider the combination with FS, where the pruning process is stopped at a certain level of reduction of candidates. We demonstrate superior results regarding the savings in computation time compared to the straightforward solution, where the fast FS-equivalent method for grayscale images is applied to each band separately. Experimental results are obtained using a standard dataset for color images and a five-band multispectral image dataset.

The paper is organized as follows: In Section 2, we provide the problem setting and required techniques as preliminaries. We present the design of 3D-OTSHT for three-dimensional (3D) integral image and the 3D block matching using 3D-OTSHT in Section 3. Our evaluations of the pruning performance and speedup are detailed in Section 4. Finally, in Section 5, we conclude our study.

## 2. Preliminaries

First, the problem targeted in this paper is stated. Next, a couple of techniques required for the proposed method are briefly described.

### 2.1. Problem Statement

Consider the problem of searching for patches similar to a given query in a multichannel image. In the full search (FS) algorithm, the matching patches are detected with a threshold in a sliding window manner by measuring the similarity of all the candidates in the whole search space. Generally, the sum of squared differences (SSD) of all the intensities in a candidate is used as the similarity.

Let q(x,y,z) be a query patch of size N×N having *B* bands, and I(u,v,w) measure an image of size M×M having *B* bands. The SSD of all the intensities of all the candidates is calculated, for 0≤u<M−N, 0≤v<M−N, and 0≤w<B, as

(1) $$\begin{array}{c} SSD\left(u,v,w\right)=\sum_{u=0}^{M-N}\sum_{v=0}^{M-N}\sum_{x=0}^{N-1}\sum_{y=0}^{N-1}\sum_{z=0}^{B-1}{\left(I(u+x,v+y,w+z)-q(x,y,z)\right)}^{2}. \end{array}$$

It turns out that ($2{\left(M-N+1\right)}^{2}N^{2}B-1$) additions and ${\left(M-N+1\right)}^{2}N^{2}B$ multiplications are required for the search. The aim of this paper is to reduce the computational complexity for search in multichannel images while keeping the same accuracy as FS using the same threshold as FS uses.

A part of I(u,v,w), i.e., the *i*-th candidate, and a query are hereafter simply expressed as $p_{i}$ and q, respectively, in a vector form, e.g., the SSD of the *i*-th candidate is represented as

(2) $$\begin{array}{c} SSD_{i}=\left|\right|p_{i}-q{\left|\right|}_{2}^{2}. \end{array}$$

### 2.2. Tree-Structured Haar Transform

Tree-structured Haar transform (TSHT) is a generalization of the Haar transform, which can be applied to signals with arbitrary length [25]. In the conventional Haar transform [26], the basis is built by dividing an interval equally into two intervals. In TSHT, on the other hand, the basis is built by dividing an interval unequally into two intervals putting weights on them. The complete division of the intervals can be expressed by a binary tree structure.

**Figure 1:**
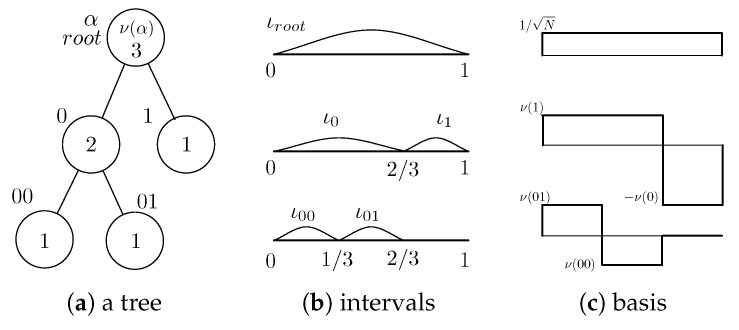
Binary tree and corresponding intervals for the basis. (**a**) Binary tree having N=3 leaves, where each node is labeled with 0 and 1 outside the circle except for root; (**b**) intervals corresponding to the nodes; (**c**) the basis built from the intervals.

Figure 1a shows an example of a binary tree having 3 leaves. A circle represents a node. The topmost node is the root. The node having no connection below (no child node) is a leaf. The number inside a circle represents the number of leaves the node has, which determines the internal division ratio when dividing an interval and the weight of intervals in the basis function. Figure 1b shows the intervals corresponding to the nodes of the binary tree. The interval corresponding to a node is divided internally in two subintervals, in a ratio equal to the number of leaves of the left child node to that of the right child node.

Let α be a node of a binary tree having *N* leaves. Let $\alpha_{0}$ and $\alpha_{1}$ be the left child node and the right child node of α, respectively. We denote by $\iota_{\alpha }$ the interval corresponding to α. The basis function for interval $\iota_{root}$ is given as

(3) $$\begin{array}{cc} h(t) & =\begin{array}{cc} \frac{1}{\sqrt{N}}, & t\in \iota_{root} \end{array} \end{array}$$

and

(4) $$\begin{array}{cc} h(t) & =\left\{\begin{array}{cc} \nu (\alpha_{1}), & t\in \iota_{\alpha_{0}}\\ -\nu (\alpha_{0}), & t\in \iota_{\alpha_{1}}\\ 0, & \mathrm{otherwise}, \end{array}\right. \end{array}$$

where ν(α) represents the number of leaves that α has. Thus, except for $\iota_{root}$, the absolute value of the weight of two intervals is inversely proportional to the number of leaves. Figure 1c shows the basis.

### 2.3. Three-Dimensional Integral Image

The three-dimensional (3D) integral image J(x,y,z) is generated from an image, I(u,v,w), of size M×M having *B* bands, for x=0,1,2,…,M; y=0,1,2,…,M; and z=0,1,2,…,B, as

(5) $$\begin{array}{c} J\left(x,y,z\right)=\sum_{u=0}^{x-1}\sum_{v=0}^{y-1}\sum_{w=0}^{z-1}I\left(u,v,w\right), \end{array}$$

where J(0,y,z)=0, J(x,0,z)=0, and J(x,y,0)=0. Observe that $\left(3B-1\right)M^{2}$ additions are required to build a 3D integral image.

**Figure 2:**
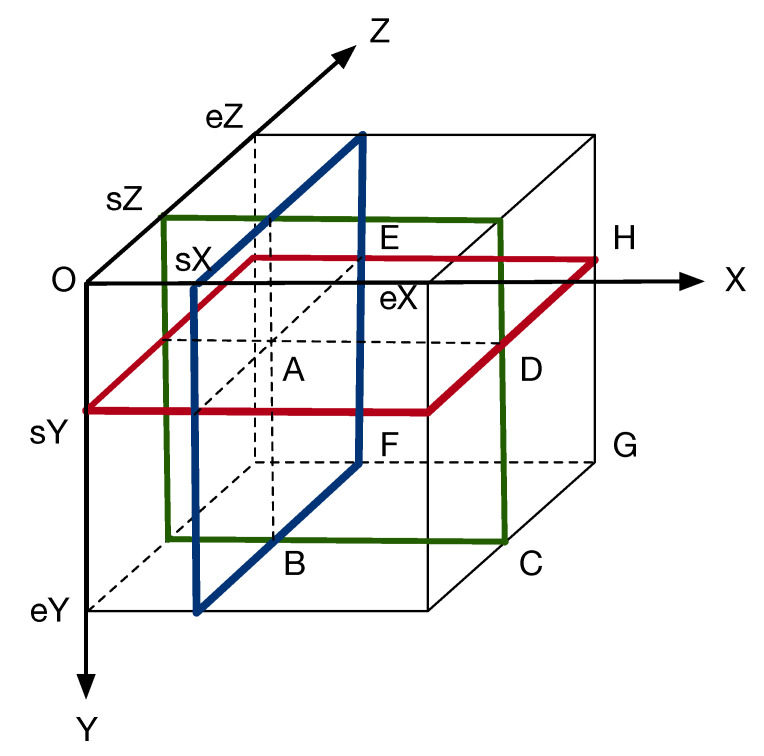
Specific property of 3D integral image. The sum of the intensities in region ABCD-EFGH whose diagonal starts at A:(sX,sY,sZ) and ends at G:(eX,eY,eZ) is obtained by seven additions.

With the 3D integral image, the sum of all the intensities in region *A* (ABCD-EFGH) whose diagonal starts at the location (sX,sY,sZ) and ends at (eX,eY,eZ), as shown in Figure 2, is calculated by seven additions regardless of region size as

(6) $$\begin{array}{cc} & regionSum(A)=J(eX+1,eY+1,eZ+1)-J(eX+1,eY+1,sZ)-J(eX+1,sY,eZ+1)\\ \; & \;\;\;\;\;\;\;\;\;\;\;-J(sX,eY+1,eZ+1)+J(sX,sY,eZ+1)+J(sX,eY+1,sZ)+J(eX+1,sY,sZ)\\ \; & \;\;\;\;\;\;\;\;\;\;\;-J(sX,sY,sZ). \end{array}$$

This property is a key to significant speedup of the proposed method.

## 3. Three-Dimensional Block Matching for Multichannel Images

We propose here fast FS-equivalent three-dimensional (3D) block matching for multichannel image using three-dimensional orthonormal tree-structured Haar transform (3D-OTSHT). First, we construct 3D-OTSHT to simplify the computation to obtain its coefficients. Next, we describe the 3D block matching using 3D-OTSHT.

### 3.1. Three-Dimensional Orthonormal Tree-Structured Haar Transform

One of the vectors forming the basis of the vector space for a three-dimensional region is referred to in this paper as a basis block. The 3D-OTSHT consists of the basis blocks built by subdividing a three-dimensional region formed by intervals along *X*, *Y*, and *Z* axis. For rapid calculation of 3D-OTSHT coefficients via integral image, we design the basis block to have at most two regions in it, each of which is assigned to a constant.

Let us consider the basis blocks for a query of size N×N having *B* bands. We generate the set of basis blocks by basis block functions. Let $T_{X}$, $T_{Y}$, and $T_{Z}$ be the binary trees for *X* axis having *N* leaves, *Y* axis having *N* leaves, and *Z* axis having *B* leaves, respectively. The nodes of $T_{X}$, $T_{Y}$, and $T_{Z}$ are denoted by α, β, and γ, respectively.

We define the basis block function for region $\left(\iota_{root}\times \iota_{root}\times \iota_{root}\right)$ as

(7) $$\begin{array}{cc} \varphi_{0}\left(x,y,z\right) & =\begin{array}{cc} \frac{1}{N\sqrt{B}}, & \left(x,y,z\right)\in \iota_{root}\times \iota_{root}\times \iota_{root} \end{array} \end{array}$$

and the basis block functions for the other regions as

(8) $$\begin{array}{cc} \varphi_{1}\left(x,y,z\right) & =\left\{\begin{array}{cc} c_{\varphi 1}^{+}, & \left(x,y,z\right)\in \iota_{\alpha_{0}}\times \iota_{\beta }\times \iota_{\gamma }\\ c_{\varphi 1}^{-}, & \left(x,y,z\right)\in \iota_{\alpha_{1}}\times \iota_{\beta }\times \iota_{\gamma }\\ 0, & \mathrm{otherwise} \end{array}\right. \end{array}$$

(9) $$\begin{array}{cc} \varphi_{2}\left(x,y,z\right) & =\left\{\begin{array}{cc} c_{\varphi 2}^{+}, & \left(x,y,z\right)\in \iota_{\alpha_{0}}\times \iota_{\beta_{0}}\times \iota_{\gamma }\\ c_{\varphi 2}^{-}, & \left(x,y,z\right)\in \iota_{\alpha_{0}}\times \iota_{\beta_{1}}\times \iota_{\gamma }\\ 0, & \mathrm{otherwise} \end{array}\right. \end{array}$$

(10) $$\begin{array}{cc} \varphi_{3}\left(x,y,z\right) & =\left\{\begin{array}{cc} c_{\varphi 3}^{+}, & \left(x,y,z\right)\in \iota_{\alpha_{1}}\times \iota_{\beta_{0}}\times \iota_{\gamma }\\ c_{\varphi 3}^{-}, & \left(x,y,z\right)\in \iota_{\alpha_{1}}\times \iota_{\beta_{1}}\times \iota_{\gamma }\\ 0, & \mathrm{otherwise} \end{array}\right. \end{array}$$

(11) $$\begin{array}{cc} \varphi_{4}\left(x,y,z\right) & =\left\{\begin{array}{cc} c_{\varphi 4}^{+}, & \left(x,y,z\right)\in \iota_{\alpha_{0}}\times \iota_{\beta_{0}}\times \iota_{\gamma_{0}}\\ c_{\varphi 4}^{-}, & \left(x,y,z\right)\in \iota_{\alpha_{0}}\times \iota_{\beta_{0}}\times \iota_{\gamma_{1}}\\ 0, & \mathrm{otherwise} \end{array}\right. \end{array}$$

(12) $$\begin{array}{cc} \varphi_{5}\left(x,y,z\right) & =\left\{\begin{array}{cc} c_{\varphi 5}^{+}, & \left(x,y,z\right)\in \iota_{\alpha_{0}}\times \iota_{\beta_{1}}\times \iota_{\gamma_{0}}\\ c_{\varphi 5}^{-}, & \left(x,y,z\right)\in \iota_{\alpha_{0}}\times \iota_{\beta_{1}}\times \iota_{\gamma_{1}}\\ 0, & \mathrm{otherwise} \end{array}\right. \end{array}$$

(13) $$\begin{array}{cc} \varphi_{6}\left(x,y,z\right) & =\left\{\begin{array}{cc} c_{\varphi 6}^{+}, & \left(x,y,z\right)\in \iota_{\alpha_{1}}\times \iota_{\beta_{0}}\times \iota_{\gamma_{0}}\\ c_{\varphi 6}^{-}, & \left(x,y,z\right)\in \iota_{\alpha_{1}}\times \iota_{\beta_{0}}\times \iota_{\gamma_{1}}\\ 0, & \mathrm{otherwise} \end{array}\right. \end{array}$$

(14) $$\begin{array}{cc} \varphi_{7}\left(x,y,z\right) & =\left\{\begin{array}{cc} c_{\varphi 7}^{+}, & \left(x,y,z\right)\in \iota_{\alpha_{1}}\times \iota_{\beta_{1}}\times \iota_{\gamma_{0}}\\ c_{\varphi 7}^{-}, & \left(x,y,z\right)\in \iota_{\alpha_{1}}\times \iota_{\beta_{1}}\times \iota_{\gamma_{1}}\\ 0, & \mathrm{otherwise}, \end{array}\right. \end{array}$$

where $c_{\varphi n}^{+}$ and $c_{\varphi n}^{-}$, (n=1,2,…,7) are a positive constant and a negative constant, respectively, which includes normalization factor and weight:

(15) $$\begin{array}{cc} c_{\varphi 1}^{+} & =\frac{\nu (\alpha_{1})}{\sqrt{\nu \left(\alpha \right)\nu \left(\beta \right)\nu \left(\gamma \right)\nu \left(\alpha_{0}\right)\nu \left(\alpha_{1}\right)}},\;\;c_{\varphi 1}^{-}=-\frac{\nu (\alpha_{0})}{\sqrt{\nu \left(\alpha \right)\nu \left(\beta \right)\nu \left(\gamma \right)\nu \left(\alpha_{0}\right)\nu \left(\alpha_{1}\right)}}, \end{array}$$

(16) $$\begin{array}{cc} c_{\varphi 2}^{+} & =\frac{\nu (\beta_{1})}{\sqrt{\nu \left(\alpha_{0}\right)\nu \left(\beta \right)\nu \left(\gamma \right)\nu \left(\beta_{0}\right)\nu \left(\beta_{1}\right)}},\;\;c_{\varphi 2}^{-}=-\frac{\nu (\beta_{0})}{\sqrt{\nu \left(\alpha_{0}\right)\nu \left(\beta \right)\nu \left(\gamma \right)\nu \left(\beta_{0}\right)\nu \left(\beta_{1}\right)}}, \end{array}$$

(17) $$\begin{array}{cc} c_{\varphi 3}^{+} & =\frac{\nu (\beta_{1})}{\sqrt{\nu \left(\alpha_{1}\right)\nu \left(\beta \right)\nu \left(\gamma \right)\nu \left(\beta_{0}\right)\nu \left(\beta_{1}\right)}},\;\;c_{\varphi 3}^{-}=-\frac{\nu (\beta_{0})}{\sqrt{\nu \left(\alpha_{1}\right)\nu \left(\beta \right)\nu \left(\gamma \right)\nu \left(\beta_{0}\right)\nu \left(\beta_{1}\right)}}, \end{array}$$

(18) $$\begin{array}{cc} c_{\varphi 4}^{+} & =\frac{\nu (\gamma_{1})}{\sqrt{\nu \left(\alpha_{0}\right)\nu \left(\beta_{0}\right)\nu \left(\gamma \right)\nu \left(\gamma_{0}\right)\nu \left(\gamma_{1}\right)}},\;\;c_{\varphi 4}^{-}=-\frac{\nu (\gamma_{0})}{\sqrt{\nu \left(\alpha_{0}\right)\nu \left(\beta_{0}\right)\nu \left(\gamma \right)\nu \left(\gamma_{0}\right)\nu \left(\gamma_{1}\right)}}, \end{array}$$

(19) $$\begin{array}{cc} c_{\varphi 5}^{+} & =\frac{\nu (\gamma_{1})}{\sqrt{\nu \left(\alpha_{0}\right)\nu \left(\beta_{1}\right)\nu \left(\gamma \right)\nu \left(\gamma_{0}\right)\nu \left(\gamma_{1}\right)}},\;\;c_{\varphi 5}^{-}=-\frac{\nu (\gamma_{0})}{\sqrt{\nu \left(\alpha_{0}\right)\nu \left(\beta_{1}\right)\nu \left(\gamma \right)\nu \left(\gamma_{0}\right)\nu \left(\gamma_{1}\right)}}, \end{array}$$

(20) $$\begin{array}{cc} c_{\varphi 6}^{+} & =\frac{\nu (\gamma_{1})}{\sqrt{\nu \left(\alpha_{1}\right)\nu \left(\beta_{0}\right)\nu \left(\gamma \right)\nu \left(\gamma_{0}\right)\nu \left(\gamma_{1}\right)}},\;\;c_{\varphi 6}^{-}=-\frac{\nu (\gamma_{0})}{\sqrt{\nu \left(\alpha_{1}\right)\nu \left(\beta_{0}\right)\nu \left(\gamma \right)\nu \left(\gamma_{0}\right)\nu \left(\gamma_{1}\right)}}, \end{array}$$

(21) $$\begin{array}{cc} c_{\varphi 7}^{+} & =\frac{\nu (\gamma_{1})}{\sqrt{\nu \left(\alpha_{1}\right)\nu \left(\beta_{1}\right)\nu \left(\gamma \right)\nu \left(\gamma_{0}\right)\nu \left(\gamma_{1}\right)}},\;\;c_{\varphi 7}^{-}=-\frac{\nu (\gamma_{0})}{\sqrt{\nu \left(\alpha_{1}\right)\nu \left(\beta_{1}\right)\nu \left(\gamma \right)\nu \left(\gamma_{0}\right)\nu \left(\gamma_{1}\right)}}. \end{array}$$

A series of functions (8) to (14) is repeated at all the intervals corresponding to the nodes of three binary trees. Eventually, $N^{2}B$ basis blocks are generated. Each basis block is indicated as $G_{k}$, ($k=1,2,\ldots ,N^{2}B$) in a vector form. As such, $c_{\varphi n}^{+}$ and $c_{\varphi n}^{-}$ are replaced by $c_{k}^{+}$ and $c_{k}^{-}$, respectively. Let G be the set of basis blocks, i.e., $G={\left[G_{1},G_{2},\ldots ,G_{N^{2}B}\right]}^{T}$, where T denotes the transposition. G is orthonormal.

**Figure 3:**
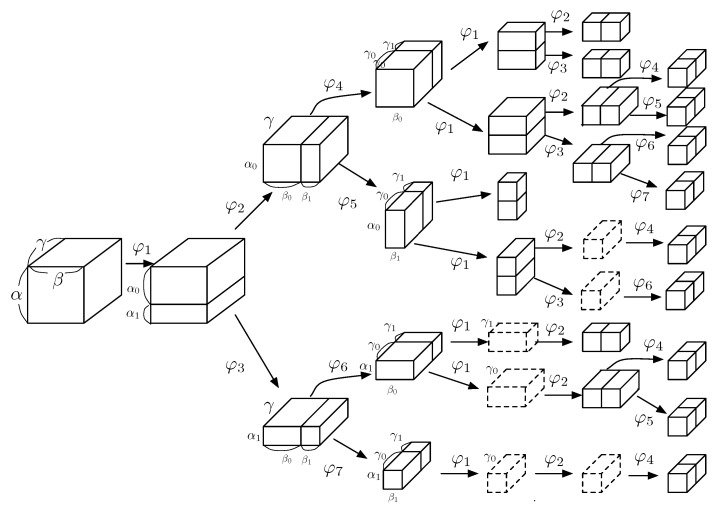
Three-dimensional orthonormal tree-structured Haar transform (3D-OTSHT) basis blocks built by subdivision.

Figure 3 shows the appearance of 3D-OTSHT. Function $\varphi_{1}$ divides a region along *X* axis into two regions, $c_{*}^{+}$ and $c_{*}^{-}$, where $c_{*}^{+}$ and $c_{*}^{-}$ are the region assigned to $c_{\varphi n}^{+}$ and $c_{\varphi n}^{-}$ as in (15) through (21), respectively. Functions $\varphi_{2}$ and $\varphi_{3}$ divide $c_{*}^{+}$ and $c_{*}^{-}$, respectively, built by $\varphi_{1}$ along *Y* axis; $\varphi_{4}$ and $\varphi_{5}$ divide $c_{*}^{+}$ and $c_{*}^{-}$, respectively, built by $\varphi_{2}$ along *Z* axis; $\varphi_{6}$ and $\varphi_{7}$ divide $c_{*}^{+}$ and $c_{*}^{-}$, respectively, built by $\varphi_{3}$ along *Z* axis.

### 3.2. 3D Block Matching Using 3D-OTSHT with 3D Integral Image

In the proposed 3D block matching, SSD of 3D-OTSHT coefficients is calculated as similarity, but not all the transform coefficients of all the candidates are used. The candidate that does not match is rejected from the search in the middle of processing, which is called pruning.

The transform coefficients are obtained efficiently with a 3D integral image. From (6), the *k*-th 3D-OTSHT coefficient, P(k), of a patch, p, in a vector form is obtained as

(22) $$\begin{array}{cc} P(k) & =G_{k}p\\ \; & =c_{k}^{+}\times regionSum\left(p_{*}^{+}\right)+c_{k}^{-}\times regionSum\left(p_{*}^{-}\right), \end{array}$$

where $p_{*}^{+}$ and $p_{*}^{-}$ are the regions in the patch corresponding to the regions to which $c_{k}^{+}$ and $c_{k}^{-}$ are assigned in the *k*-th basis block, respectively.

For pruning, the similarity of candidates is calculated using a subset of 3D-OTSHT. Let $G^{k}$ be the subset of G that contains the first *k* basis blocks, i.e., $G^{k}={\left[G_{1},G_{2},\ldots ,G_{k}\right]}^{T}$. The similarity of the *i*-th candidate is calculated with $G^{k}$ as

(23) $$\begin{array}{c} SSD_{i}^{k}=\left|\right|P_{i}^{k}-Q^{k}{\left|\right|}_{2}^{2}, \end{array}$$

where $P_{i}^{k}=G^{k}p_{i}$ and $Q^{k}=G^{k}q$. At every *k* ($k=1,2,\ldots ,N^{2}B$), $SSD_{i}^{k}$ is judged with a threshold. Once $SSD_{i}^{k}$ is beyond the threshold, the *i*-th candidate is rejected from the search and neither OTSHT coefficient nor SSD is calculated afterward. As long as using the same threshold as FS uses, the unmatched candidates are securely rejected. The theory behind this is that if the transform is orthonormal, the energy of a signal is not changed in the transformed domain. Therefore, it holds that

(24) $$\begin{array}{c} \left|\right|P_{i}-{Q||}_{2}^{2}=\left|\right|p_{i}-q{\left|\right|}_{2}^{2}, \end{array}$$

where $P_{i}=Gp_{i}$ and Q=Gq. From $\left|\right|P_{i}^{k}-Q^{k}{\left|\right|}_{2}^{2}\leq \left|\right|P_{i}-Q{\left|\right|}_{2}^{2}$ for $k=1,2,\ldots ,N^{2}B$, secure rejection is made. For this reason, the transform should be orthonormal and SSD used as the similarity measure.

### 3.3. 3D Block Matching Using Limited 3D-OTSHT

All the basis blocks of 3D-OTSHT can detect patches with the same accuracy as FS. However, it is inefficient to use all the basis blocks because $G_{k}$ becomes sparser as *k* increases. To avoid this, the limited number of basis blocks is used for pruning, and after the number of candidates is reduced, the remaining candidates are scrutinized by FS. That is, $G^{K}$ with a certain *K* is used instead of G for $SSD_{i}^{k}$ shown in (23), and at every *k*, (k=1,2,…,K), $SSD_{i}^{k}$ is judged with a threshold for pruning.

## 4. Evaluation

We performed 3D block matching in order to evaluate the pruning performance and elapsed time of the proposed method using multichannel images.

### 4.1. Methods and Environments

We compared the performance of the following five methods for search: FS, two-dimensional OTSHT with a two-dimensional integral image by single judge (2D-OTSHT-2DI-S) [24], two-dimensional OTSHT with two-dimensional integral image by whole judge (2D-OTSHT-2DI-W), two-dimensional OTSHT with a 3D integral image (2D-OTSHT-3DI) [27], and the proposed 3D-OTSHT with 3D integral image (3D-OTSHT-3DI). 2D-OTSHT-2DI-S and -W use the OTSHT for grayscale images on each channel, and judge the candidates in different ways: The single judge (-S) decides whether to reject the candidate or not based on a single channel, while the whole judge (-W) evaluates the candidate based on all the channels. In 2D-OTSHT-3DI, a basis image forms a basis block for the 3D integral image so that the same basis image is piled up.

We use two image data sets, color image data set and five-band multispectral image data set. The color image data set, called SIDBA, contains 12 scenes of size 256 × 256 having 3 color channels [28]. The 5-band image data set contains 11 scenes of size 1824 × 1368 having 5 bands [29]. For each dataset, five patch sizes are used. We chose 10 queries randomly in an image per patch size. Then, we obtain the ground truth patches similar to the query by FS with a threshold. If the SSD of the *i*-th patch holds

(25) $$\begin{array}{c} \left|\right|p_{i}-q{\left|\right|}_{2}^{2}<=threshold, \end{array}$$

we set the *i*-th patch a ground truth. In this experiment, the threshold is $10N^{2}B$ for the color images and $2N^{2}B$ for the 5-band multispectral images. The same threshold is used in all the methods. Table 1 summarizes the number of ground truth patches. From Table 1, it can be seen that the mean number of ground truth patches tends to decrease as the patch size increases and increase as the image size increases. We confirmed that the number of ground truth patches chosen by the queries with low standard deviations of pixel intensities is likely to be large, and that the number of ground truth patches chosen by the queries with high standard deviations is likely to be one. Therefore, the threshold should be set appropriately considering the size and characteristics of images in practical applications. Generally, the larger threshold yields more ground truth patches. However, a threshold that is too large will be meaningless.

All the algorithms are written in C as single thread tasks, compiled with Xcode 10.1, and run on a macOS system with 4 GHz Intel core i7 and 16 GB RAM, where eight active processor cores with hardware multithreading are used.

### 4.2. Pruning Performance

The pruning performance is evaluated by the ratio, R(K), of the number of remaining extra patches detected by *K* basis images/blocks to the number of all the candidates, which is defined by

(26) $$\begin{array}{c} R\left(K\right)=\frac{N_{d}\left(K\right)-N_{g}}{N_{c}}\times 100, \end{array}$$

where $N_{d}\left(K\right)$ refers to the number of patches detected by *K* basis images/blocks, $N_{g}$ is the number of ground truth patches, and $N_{c}$ represents the number of all the candidates. Lower R(K) means better performance.

**Figure 4:**
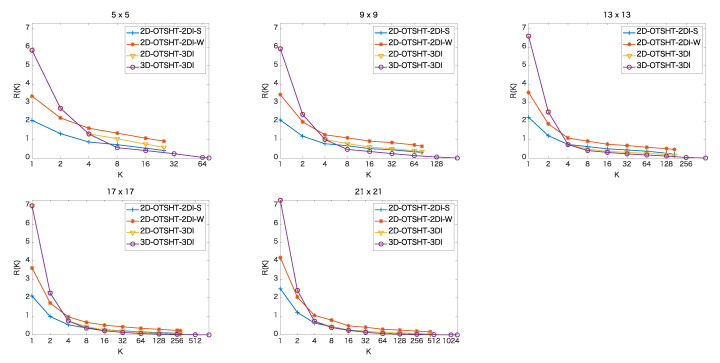
Percentage of extra patches remaining at *K* basis images/blocks in color images of size 256×256.

**Figure 5:**
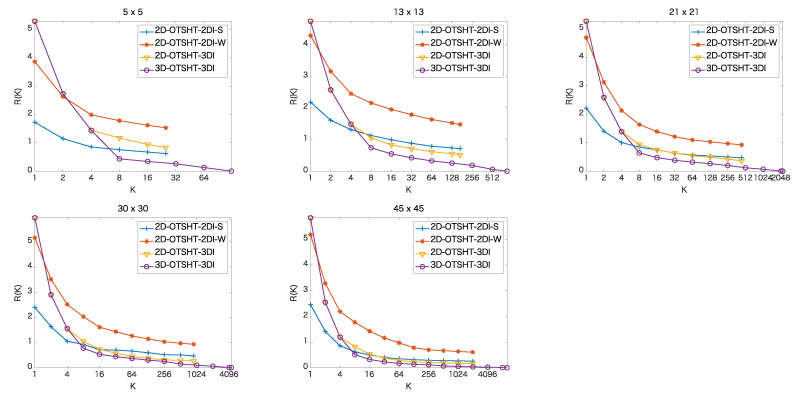
Percentage of extra patches remaining at *K* basis images/blocks in 5-band images of size 1824×1368.

Figure 4 and Figure 5 show the pruning performance, R(K), ($K=2,4,8,\ldots ,K_{max}$), in the color images and the 5-band multispectral images, respectively, where $K_{max}$ refers to the maximum number of basis images/blocks. In the proposed 3D-OTSHT-3DI, $K_{max}$ = $N^{2}B$, while in the other methods, $K_{max}$ = $N^{2}$. We confirmed that all the methods detect every ground truth patch at any *K*, i.e., there are no false negatives. Therefore, the final accuracy (the accuracy after the remaining candidates are scrutinized by FS) is the same as the accuracy of FS. The proposed 3D-OTSHT-3DI method yields the best pruning performance both on 3-band and 5-band images for all the patch sizes. In both image data sets, we observe that when *K* is greater than or equal to 8, 3D-OTSHT-3DI is better than the other methods and that as *K* increases, the rate of change reduces. These facts suggest that not all the basis blocks are required for the whole speedup.

**Figure 6:**
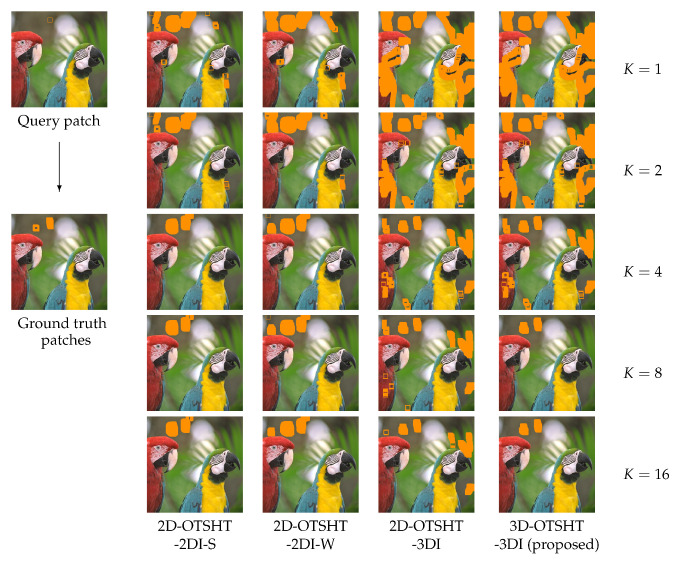
An example of the patches detected by a query patch in different methods at *K* basis images/blocks. The query patch of size 13×13 and the detected patches are shown in orange squares.

Figure 6 shows an example of patches detected at *K* basis images/blocks by different methods and for different values of *K*. Every one of the detected patches of size 13 × 13 is shown in an orange square, and the detected overlapping patches cover different areas depending on the employed method and the *K*-value. It can be seen that the amount of candidate patches reduces as *K* increases. The result of 3D-OTHST-3DI does not differ significantly between K=8 and K=16 and, clearly, these results agree best with the ground truth.

### 4.3. Computational Complexity

Here, we consider the number of arithmetic operations with respect to the number of basis images/blocks when we use only OTSHT not included in the arithmetic operations of FS. Table 2 shows the number of additions and multiplications per pixel for search for patches similar to a query of size N×N having *B* bands including building an integral image. It shows the worst case, where no candidates are rejected at any *K* basis images/blocks. In 2D-OTSHT-2DI-S and -W, two additions are needed per pixel for building a 2D-integral image in each band, and (8K−1) additions and 3K multiplications are required per pixel for SSD in each band. Thus, in total, (2+8k−1)B additions and 3KB multiplications per pixel. In 2D- and 3D-OTSHT-3DI, (3B−1) additions are needed per pixel for a 3D-integral image, and (16K−1) additions and 3K multiplications are required per pixel for SSD. For the same *K*, the number of additions for 2D- and 3D-OTSHT-3DI is smaller than that for 2D-OTSHT-2DI-S and -W, except for the images having 2 bands; while the number of multiplications for 2D- and 3D-OTSHT-3DI is smaller than that for 2D-OTSHT-2DI-S and -W in any images with more than one band.

### 4.4. Speedup

**Figure 7:**
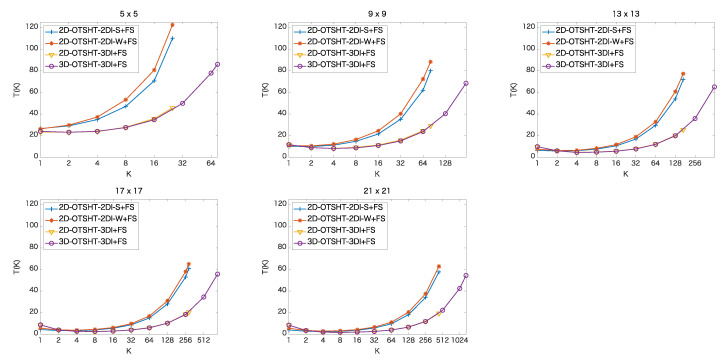
Mean ratio [%] of the elapsed time of each method at *K* basis images/blocks to the elapsed time of FS in color images of size 256 × 256.

**Figure 8:**
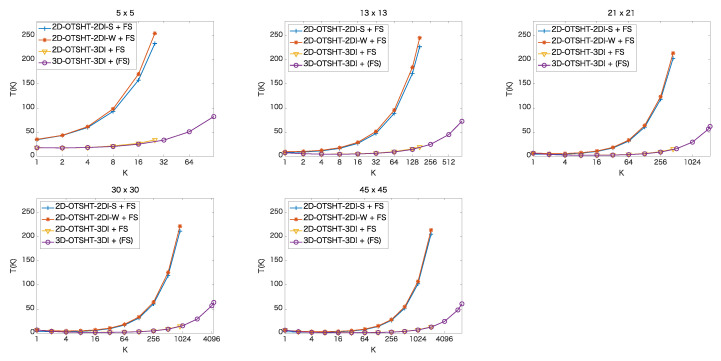
Mean ratio [%] of the elapsed time of each method at *K* basis images/blocks to the elapsed time of FS in 5-band images of size 1824 × 1368.

In this section, we analyze when to stop the pruning process for the whole speedup in combination with FS, showing the mean elapsed time at *K* basis images/blocks in the methods, and compare the final performance of the five methods. Figure 7 and Figure 8 show the mean ratio (%) of the elapsed time of each method at *K* basis images/blocks to the elapsed time of FS. Table 3 and Table 4 detail the mean elapsed time and the ratio to the elapsed time of FS when we use $G^{K}$, (K=1,2,4,…,32) for the color images and the 5-band multispectral images, respectively. The fastest time at each patch size is expressed in bold. From Table 3 and Table 4, it can be seen that the proposed method outperforms the other methods except for the cases of patches of size 9×9 in the color images and patches of size 5×5 in the 5-band images. In the color images, for patch size 5×5, the fastest mean elapsed time was 1.03 (ms) which is 23.17 % of the mean elapsed time of FS; while for 21×21, the fastest mean elapsed time was 1.30 (ms), which is 1.78 % of the mean elapsed time of FS. In the 5-band images, for patch size 5×5, the fastest mean elapsed time was 0.050 (s) which is 17.44 % of the mean elapsed time of FS; while for 45×45, the fastest mean elapsed time was 0.164 (s), which is 0.78 % of the mean elapsed time of FS. The larger the patch size and the larger the number of bands, the higher the efficiency of the proposed method.

We confirmed that the mean ratio did not differ much between eight active processor cores with hardware multithreading and one core in the same system.

We also compared the five methods with the best performed *K*-value to A2DHT [20]. A2DHT is an FS-equivalent algorithm for grayscale images and reportedly performs fastest in FS-equivalent algorithms, whose source code is provided by the authors [30]. We modified the code so that the method was applied to each band separately. Since A2DHT has a limitation that the patch size is power-of-2, the queries of size 16×16 were used for the color image dataset and the queries of size 16×16 and 32×32 were used for the 5-band image dataset. The number of ground truth patches is summarized in Table 5. We confirmed that all the methods detected every ground truth patch. Table 6 shows the mean elapsed time and mean ratio to FS time, where the fastest time and its ratio at each patch size is expressed in bold. It can be seen that the proposed method outperforms state-of-the-art methods.

## 5. Conclusions

We proposed a fast FS-equivalent 3D block matching method in order to search for the patch(es) similar to a given query for multichannel images. The proposed method uses 3D-OTSHT and reduces the number of candidates with SSD in the transformed domain. The pruning process rejects unmatched candidates during the block matching processing. Moreover, in combination with FS, the pruning process is stopped during the processing for the whole speedup. We designed 3D-OTSHT making the most of 3D integral image rather than the extension of one-dimensional TSHT. Unmatched patches are securely rejected due to being orthonormal and using SSD as the similarity measure. We have analyzed the pruning performance and mean elapsed time using a color image dataset and a 5-band multispectral image dataset, and demonstrated that the proposed method outperforms state-of-the-art methods. In the color images, the mean elapsed time was shortened up to less than 2% of the FS time. In the 5-band multispectral images, the search time was shortened up to around 0.8% of the FS time, hence allowing more than 100-times faster processing without sacrificing the accuracy. We believe that these huge savings in computation time can enable new applications of patch matching in multichannel images, which were not feasible before due to the prohibitive computational complexity.

## Figures and Tables

**Table 1:** The number of ground truth patches.

						Number of Patches		
Data Set	Image Size	Band	Scenes	Patch Size	Samples	Min.	Mean	Max.
				5×5	120	1	265	7541
				9×9	120	1	308	6097
1	256×256	3	12	13×13	120	1	234	4937
				17×17	120	1	51	1564
				21×21	120	1	4	127
				5×5	110	1	6158	103,161
				13×13	110	1	5634	113,573
2	1824×1368	5	11	21×21	110	1	2508	89,534
				30×30	110	1	1543	85,131
				45×45	110	1	1882	74,967

**Table 2:** The number of additions and multiplications per pixel for searching patches having *B* bands with *K* basis images/blocks.

Method	Additions	Multiplications
2D-OTSHT-2DI-S and -W	(2+8K−1)B	3KB
2D- and 3D-OTSHT-3DI	3B−1+16K−1	3K

**Table 3:** Mean elapsed time and ratio to full search (FS) time in color images of size 256 × 256.

			2D-OTSHT-2DI		2D-OTSHT-2DI		2D-OTSHT-3DI		3D-OTSHT-3DI	
	FS		-S+FS [24]		-W+FS		+FS [27]		+FS (Proposed)	
Size	[ms]	K	Time [ms]	Ratio [%]	Time [ms]	Ratio [%]	Time [ms]	Ratio [%]	Time [ms]	Ratio [%]
		1	1.18	26.57	1.17	26.26	1.04	23.23	1.07	23.98
5		2	1.30	29.07	1.33	29.78	1.03	23.18	**1.03**	**23.17**
×	4.46	4	1.55	34.89	1.66	37.21	1.06	23.79	1.07	24.00
5		8	2.10	47.12	2.38	53.30	1.24	27.78	1.23	27.53
		16	3.15	70.77	3.60	80.71	1.59	35.75	1.55	34.71
		32	—	—	—	—	—	—	2.23	49.96
		1	1.46	10.07	1.55	10.67	1.68	11.60	1.70	11.72
9		2	1.44	9.92	1.53	10.54	1.29	8.91	1.29	8.87
×	14.51	4	1.62	11.18	1.76	12.12	**1.18**	**8.14**	1.19	8.18
9		8	2.14	14.74	2.38	16.38	1.34	9.24	1.26	8.70
		16	3.13	21.58	3.55	24.50	1.64	11.31	1.58	10.88
		32	5.11	35.19	5.84	40.22	2.30	15.86	2.19	15.12
		1	1.82	6.39	2.11	7.43	2.77	9.76	2.78	9.80
13		2	1.64	5.77	1.77	6.24	1.71	6.03	1.70	5.97
×	28.39	4	1.72	6.06	1.83	6.45	1.29	4.54	**1.26**	**4.44**
13		8	2.14	7.53	2.35	8.29	1.38	4.85	1.34	4.70
		16	3.00	10.55	3.33	11.73	1.62	5.71	1.58	5.58
		32	4.80	16.90	5.38	18.96	2.22	7.81	2.19	7.70
		1	2.19	4.39	2.82	5.66	4.35	8.74	4.35	8.72
17		2	1.71	3.42	2.01	4.03	2.04	4.09	2.01	4.04
×	49.84	4	1.66	3.34	1.91	3.82	1.34	2.68	1.34	2.69
17		8	2.00	4.02	2.26	4.53	1.33	2.67	**1.27**	**2.55**
		16	2.78	5.58	3.09	6.19	1.51	3.04	1.46	2.93
		32	4.36	8.74	4.87	9.77	2.01	4.04	1.97	3.96
		1	2.94	4.02	4.10	5.60	6.17	8.44	6.19	8.46
21		2	2.04	2.79	2.60	3.55	2.63	3.59	2.57	3.51
×	73.16	4	1.81	2.48	2.15	2.94	1.44	1.97	1.43	1.95
21		8	2.08	2.84	2.44	3.33	1.38	1.88	**1.30**	**1.78**
		16	2.71	3.71	3.13	4.28	1.49	2.03	1.47	2.01
		32	4.24	5.80	4.87	6.66	1.95	2.66	1.90	2.60

All the algorithms are written in C as single thread tasks, compiled with Xcode 10.1, and run on a macOS system with 4 GHz Intel core i7 and 16 GB RAM, where eight active processor cores with hardware multithreading are used.

**Table 4:** Mean elapsed time and ratio to FS time in 5-band images of size 1824 × 1368.

			2D-OTSHT-2DI		2D-OTSHT-2DI		2D-OTSHT-3DI		3D-OTSHT-3DI	
	FS		-S+FS [24]		-W+FS		+FS [27]		+FS (Proposed)	
Size	[s]	K	Time [s]	Ratio [%]	Time [s]	Ratio [%]	Time [s]	Ratio [%]	Time [s]	Ratio [%]
		1	0.099	34.913	0.102	35.845	0.052	18.239	0.051	17.873
5		2	0.125	44.007	0.126	44.373	**0.050**	**17.437**	0.050	17.604
×	0.285	4	0.173	60.934	0.179	62.793	0.052	18.389	0.053	18.487
5		8	0.271	95.202	0.286	100.577	0.062	21.896	0.059	20.621
		16	0.460	161.597	0.496	174.368	0.078	27.316	0.073	25.471
		32	—	—	—	—	—	—	0.097	34.203
		1	0.137	7.792	0.169	9.646	0.126	7.192	0.125	7.141
13		2	0.153	8.742	0.176	10.065	0.092	5.250	0.092	5.231
×	1.753	4	0.196	11.160	0.217	12.361	0.077	4.392	0.077	4.401
13		8	0.288	16.453	0.314	17.910	0.078	4.473	**0.072**	**4.134**
		16	0.473	26.997	0.512	29.224	0.090	5.112	0.083	4.724
		32	0.843	48.094	0.914	52.168	0.115	6.587	0.107	6.101
		1	0.211	4.360	0.324	6.696	0.302	6.250	0.301	6.235
21		2	0.195	4.035	0.275	5.681	0.177	3.658	0.176	3.637
×	4.833	4	0.221	4.577	0.276	5.719	0.122	2.516	0.122	2.525
21		8	0.306	6.322	0.351	7.266	0.108	2.228	**0.094**	**1.939**
		16	0.483	9.983	0.529	10.936	0.111	2.299	0.098	2.034
		32	0.834	17.252	0.899	18.607	0.133	2.747	0.117	2.428
		1	0.352	3.742	0.589	6.252	0.616	6.547	0.616	6.547
30		2	0.287	3.053	0.459	4.871	0.332	3.527	0.330	3.503
×	9.415	4	0.286	3.034	0.418	4.437	0.209	2.222	0.207	2.195
30		8	0.357	3.788	0.470	4.990	0.169	1.798	0.142	1.512
		16	0.527	5.595	0.623	6.620	0.154	1.631	**0.133**	**1.407**
		32	0.874	9.284	0.985	10.460	0.163	1.730	0.147	1.559
		1	0.642	3.057	1.202	5.724	1.294	6.163	1.297	6.178
45		2	0.444	2.113	0.832	3.961	0.606	2.888	0.605	2.883
×	20.993	4	0.371	1.768	0.651	3.102	0.328	1.565	0.327	1.556
45		8	0.409	1.948	0.659	3.138	0.257	1.224	0.193	0.920
		16	0.554	2.638	0.768	3.659	0.211	1.004	**0.164**	**0.781**
		32	0.876	4.173	1.076	5.124	0.200	0.952	0.168	0.802

All the algorithms are written in C as single thread tasks, compiled with Xcode 10.1 and run on a macOS system with 4 GHz intel core i7 and 16 GB RAM, where eight active processor cores with hardware multi-threading are used.

**Table 5:** The number of ground truth patches of size power-of-2.

						Number of Patches		
Dataset	Image Size	Band	Scenes	Patch Size	Samples	Min.	Mean	Max.
1	256×256	3	12	16×16	120	1	15	732
2	1824×1368	5	11	16×16	110	1	4880	100,278
				32×32	110	1	1370	91,325

**Table 6:** Mean elapsed time and ratio to FS time for patches of size power-of-2.

	Data					2D-OTSHT		2D-OTSHT		2D-OTSHT		3D-OTSHT-3DI	
Size	Set	FS		A2DHT [20]		-2DI-S+FS [24]		-2DI-W+FS		-3DI+FS [27]		+FS (Proposed)	
		**[ms]**	**[%]**	**[ms]**	**[%]**	**[ms]**	**[%**]	**[ms]**	**[%]**	**[ms]**	**[%]**	**[ms]**	**[%]**
16 × 16	1	46.04	100	2.65	5.76	1.69	3.68	1.94	4.22	1.33	2.89	**1.23**	**2.67**
						(K=4)		(K=4)		(K=4)		(K=8)	
		[s]	[%]	[s]	[%]	[s]	[%]	[s]	[%]	[s]	[%]	[s]	[%]
16 × 16	2	2.517	100	0.099	3.933	0.136	5.398	0.166	6.614	0.069	2.728	**0.067**	**2.647**
						(K=2)		(K=2)		(K=4)		(K=8)	
32 × 32	2	10.854	100	0.124	1.142	0.246	2.262	0.342	3.155	0.120	1.105	**0.108**	**0.995**
						(K=4)		(K=4)		(K=16)		(K=16)	

All the algorithms are written in C as single thread tasks, compiled with Xcode 10.1, and run on a macOS system with 4 GHz Intel core i7 and 16 GB RAM, where eight active processor cores with hardware multithreading are used.
